# Gut Microbes Meet Machine Learning: The Next Step towards Advancing Our Understanding of the Gut Microbiome in Health and Disease

**DOI:** 10.3390/ijms24065229

**Published:** 2023-03-09

**Authors:** Mauro Giuffrè, Rita Moretti, Claudio Tiribelli

**Affiliations:** 1 Department of Medical, Surgical and Health Sciences, University of Trieste, 34149 Trieste, Italy; 2 Department of Internal Medicine, Yale School of Medicine, Yale University, New Haven, CT 06510, USA; 3 Fondazione Italiana Fegato-Onlus, The Liver-Brain Unit “Rita Moretti”, 34149 Trieste, Italy

**Keywords:** gut microbiota, gut microbiome, health, microbiome, eubiosis, dysbiosis, omics, metagenomics, machine learning, supervised learning, unsupervised learning, artificial intelligence

## Abstract

The human gut microbiome plays a crucial role in human health and has been a focus of increasing research in recent years. Omics-based methods, such as metagenomics, metatranscriptomics, and metabolomics, are commonly used to study the gut microbiome because they provide high-throughput and high-resolution data. The vast amount of data generated by these methods has led to the development of computational methods for data processing and analysis, with machine learning becoming a powerful and widely used tool in this field. Despite the promising results of machine learning-based approaches for analyzing the association between microbiota and disease, there are several unmet challenges. Small sample sizes, disproportionate label distribution, inconsistent experimental protocols, or a lack of access to relevant metadata can all contribute to a lack of reproducibility and translational application into everyday clinical practice. These pitfalls can lead to false models, resulting in misinterpretation biases for microbe–disease correlations. Recent efforts to address these challenges include the construction of human gut microbiota data repositories, improved data transparency guidelines, and more accessible machine learning frameworks; implementation of these efforts has facilitated a shift in the field from observational association studies to experimental causal inference and clinical intervention.

## 1. The Human Microbiome

In recent years, there has been a significant increase in research on gut microbiota due to the growing understanding of the critical role that gut microbiota plays in human health. The human gastrointestinal tract hosts a diverse community of microorganisms, including bacteria, archaea, fungi, microbial eukaryotes, and viruses, all of which exist in a symbiotic relationship with the human host. This collection of microbes is known as the microbiota, and their genetic material is referred to as the microbiome [1]. In the past, it was believed that the number of cells in the human microbiota was ten times greater than the number of cells in the human body. However, more recent evidence has shown that the ratio is much closer to one-to-one, with a slight advantage for our microbes [2]. The gut microbiome, comprising almost 100 trillion bacteria, has a genome 150 times larger than the human host (3 million vs. approximately 23,000 genes, respectively) [3,4]. In healthy individuals, the host and microbiome maintain a healthy balance referred to as eubiosis, which can be altered to a state of dysbiosis (i.e., an abnormal shift in microbiota compositions) found in several pathological conditions [5,6,7]. The actual association between dysbiosis and the development of disease remains largely unclear, and we expect that defining this connection will be among the greatest medical challenges of the next few decades.

## 2. Machine Learning and Gut Microbiome

Advances in technology and cost reduction have made it possible to study the previously unexplored landscape of the human gut microbiome at a large scale. Omics-based methods such as metagenomics, metatranscriptomics, and metabolomics are widely used to assess the human gut microbiota [8,9]. These techniques enable high-throughput and high-resolution studies of the overall microbial community [8,9] and approach the microbiome from multiple perspectives. For example, metagenomics techniques (e.g., 16S rRNA gene sequencing or whole-genome shotgun sequencing) provide information about the overall microbial genetic content of the community of interest, and metabolomics measure the concentrations of different compounds produced by that specific community [8,9]. The use of omics-based methods has generated a large amount of data, which has prompted the development of computational methods, such as machine learning, to aid in processing and to analyze this data related to human gut microbiota research [10,11,12]. Machine learning comprises a series of powerful computational tools that have become increasingly important in various fields, including data analysis, computer vision, natural language processing, and predictive modeling [13,14]. Machine learning is a subfield of artificial intelligence that involves the development of algorithms that can learn from data and make predictions or decisions without being explicitly programmed [13,14]. Machine learning algorithms can be divided into two main categories: supervised and unsupervised learning. Supervised learning is the most common type of machine learning, and it involves training an algorithm on a labeled dataset to predict the outcome for new, unseen data. In supervised learning, the algorithm learns to identify patterns or relationships in the data that can be used to make predictions [13,14]. Examples of supervised learning algorithms include linear regression, decision trees, and support vector machines [13,14]. Unsupervised learning is usually employed to discover patterns or structures in unlabeled data [13,14]. Unsupervised learning can help identify patterns or groups in data that may not be obvious [13,14]. Examples of unsupervised learning algorithms include clustering, dimensionality reduction, and anomaly detection [13,14]. Overall, machine learning has become a powerful tool for data analysis and outcome prediction; it can be used to identify patterns and relationships in data that may not be evident, and to make predictions that would be difficult or impossible to define using traditional methods [13,14].

Several studies that analyzed gut microbiota as a potential classifier for diseases showed that microbial features in species, genes, or metabolites could differentiate between cases and healthy subjects or even predict responses to drug treatments, as brilliantly summarized by Marcos-Zambrano et al. [12]. Nevertheless, we will provide a few notable examples in the following text.

Zeller et al. [15] developed a logistic regression model based on gut microbiome composition to discriminate colorectal cancer (CRC) patients from healthy subjects. The authors employed the least absolute shrinkage and selection operator (LASSO) method to withdraw the least informative microbial species from the final method. The authors reported that the AUC-ROC value for the diagnostic model was 0.80, indicating that the model had good performance in distinguishing between patients with CRC and healthy controls. Additionally, the diagnostic model could distinguish between early-stage and advanced-stage CRC with an AUC-ROC value of 0.78. The model demonstrated a performance comparable to that of the fecal occult blood test (FOBT). However, the sensitivity drastically improved when the model was combined with the FOBT (49% increase). Specifically, *Fusobacterium nucleatum* and *Peptostreptococcus stomatis* were identified as the most relevant species to the prediction model, as previously found in association studies between CRC and microbiota [16,17].

Derosa et al. [18] tried to determine whether the gut microbiota abundance of several species could discriminate responses to immunotherapy (nivolumab) in a cohort of patients with renal cell cancer. The authors employed partial least square discriminant analysis (PLS-DA), a supervised algorithm that combines feature extraction and discriminant analysis into one algorithm and applies well to high-dimensional data [19]. Their results highlighted that some species (*Clostridiales clostridioforme* and *Clostridiales hathewayi*) were associated with drug resistance and with cancer metastasis status. Conversely, other commensal species (*Acetobacter senegalensis* and *Akkermansia muciniphila*) were associated with favorable prognosis and increased drug response. Other studies have confirmed that *Akkermansia muciniphila* could be related to more favorable treatment responses in other cancers, such as non-small-cell lung cancer patients undergoing programmed death-1 (PD-1) immunotherapy [20,21]. These findings prove that computation methods are crucial to discovering possible microbiological signatures to improve disease diagnosis or predict therapeutic responses.

Although classifiers are often employed for predicting categorical variables, such as “healthy” vs. “disease”, regression models are more appropriate for predicting continuous variables, such as metabolite levels. In recent research, regression models have been utilized to predict metabolite levels from microbial features, such as species or genes, and applied in studies examining the association between microbes and metabolites. For instance, Reiman et al. [22] trained a multilayer perceptron (MLP) model to predict metabolite levels based on microbial abundances. The contribution of individual microbes to a given metabolite level was estimated using the weights of the MLP model. An MLP model is an artificial neural network composed of multiple layers of interconnected nodes or perceptrons [23]. These layers are typically arranged in a feedforward structure, where the input is processed through each layer sequentially, and the final layer produces the output [23]. MLP is often used for supervised learning tasks, such as classification and regression, and can be trained using various algorithms, such as backpropagation [23]. The authors found that the MLP model was more accurate at predicting metabolite abundances and identified metabolite levels better than other linear models currently used for individual metabolite predictions. Furthermore, the authors showed that the MLP model could group microbes and metabolites with similar patterns of interaction and functions, which could provide insights into the microbe–metabolite interaction network’s underlying structure and reveal uncharacterized metabolites through “guilt by association”. These findings suggest that using machine learning techniques for integrating and identifying patterns in omics data is crucial to understanding the role of microbes and microbial metabolites in disease progression.

Computational technique design is not confined to data analysis, as illustrated by a recent study that employed machine learning to build a tailored menu for a nutritional intervention trial, recently published in the prestigious *New England Journal of Medicine*. In their study, Chen et al. [24], performed a randomized controlled trial to define the effect of a microbiota-directed complementary food (MDCF) intervention to treat undernourished children by employing an analysis base on linear mixed-effects models, resulting in a significant superiority in terms of weight gain and restoration of “healthy” microbiota composition.

### 2.1. Challenges in Current Application

Despite the encouraging results of machine learning techniques for studying the relationship between microbiome and disease, significant challenges still need to be addressed. One of the most critical challenges is the dependence of supervised learning models on the quantity and quality of training data. This dependence can lead to models that lack reproducibility due to small sample sizes, disproportionate label distribution, inconsistent experimental protocols, or a lack of access to relevant metadata, which can all contribute to a lack of reproducibility [25,26]. For example, two meta-analyses found that, while dysbiosis was present in CRC patients, a particular bacterial diversity was peculiar to a given population and not present in other investigations [27,28]. Furthermore, researchers must exercise caution when implementing machine learning, particularly for supervised learning tasks, to prevent pitfalls such as information leaking from the training phase to the test phase [29]. These flaws might result in excessively optimistic models and the misperception of bias as microbe–disease correlations. In the opinion of many experts, recent initiatives aimed at addressing the issues associated with machine learning in microbiome research, including the creation of human gut microbiota data repositories [29,30,31,32,33], improved data disclosure guidelines [34], and more accessible frameworks [35,36], could lead to the development of more accurate and reliable machine learning models, providing valuable insights into the mechanisms underlying microbial dysbiosis and the potential for targeted interventions to improve human health.

### 2.2. The Importance of Data Repositories and Data Preprocessing

Combining human gut microbiome data repositories with increased transparency in data sharing allows researchers to conduct meta-analyses across various studies, which can lead to the identification of robust biomarkers or indicators of dysbiosis specific to certain diseases [27,28]. In the opinion of many researchers, the availability of preprocessed data in these repositories can minimize technical biases and lower computational costs. Still, they may require more flexibility regarding tool selection or desired output formats for the user. For instance, Pasolli et al. [30] created a curated repository of human gut microbiome data preprocessed using a unified metagenome processing pipeline (e.g., bioBakery [37]). They included whole genome shotgun metagenomic (taxonomic and functional) gene abundance profiles and curated metadata. However, certain methods may require a specific and custom input data format, making the preprocessed data more challenging or incompatible. For example, the Dirichlet multinomial mixture method [38] requires integers data as input, whereas preprocessed relative microbial or relative gene abundances cannot be used as input. Therefore, while preprocessed data repositories (e.g., MGnify [31]) offer more flexibility in downstream analyses, they require more computational resources and expertise in bioinformatics to process.

In the opinion of many researchers, the expansion of public repositories is a crucial step in enabling researchers to address formerly unknown biological topics by providing more human gut microbiome-omics data. This will likely lead to a more significant usage of machine learning techniques on an increasing amount of publicly available omics data. However, we believe that building algorithms from scratch can be time-consuming, prone to error, and not applicable to other clinical settings due to the lack of methodology standardization. Therefore, the use of a machine learning framework, which is a comprehensive collection of tools that supports the analytical process, from data preprocessing to model validation, can be significant in avoiding the most common machine learning errors, making the analysis more efficient and robust. Several machine learning frameworks are available, each written in a different programming language and featuring a variety of modeling techniques; some of these are specifically tailored for microbiome data. From our perspective, the increased accessibility and repeatability of human gut microbiome investigations provided by these frameworks are essential in lowering the danger of overfitting. An excellent example is represented by the framework developed by Topçuoğlu et al. [39], who trained seven models using fecal 16S rRNA sequence data to predict the presence of CRC by creating a reusable, open-source pipeline able to train, validate, and interpret these models. Various machine learning approaches, including logistic regression, support vector machines, decision trees, and random forests, were examined in terms of performance, interpretability, and training time. The logistic regression model was simple, rapid, and interpretable, whereas the random forest model performed best in detecting CRC, but still was difficult to train. Their findings emphasize the need to select a methodological strategy aligned with the study’s aims to balance performance and interpretability. From our point of view, the application of machine learning frameworks has the potential to revolutionize the actual state of the art, but researchers must exercise caution and choose appropriate methodology for their specific research questions and goals.

## 3. Conclusions

**Figure 1:**
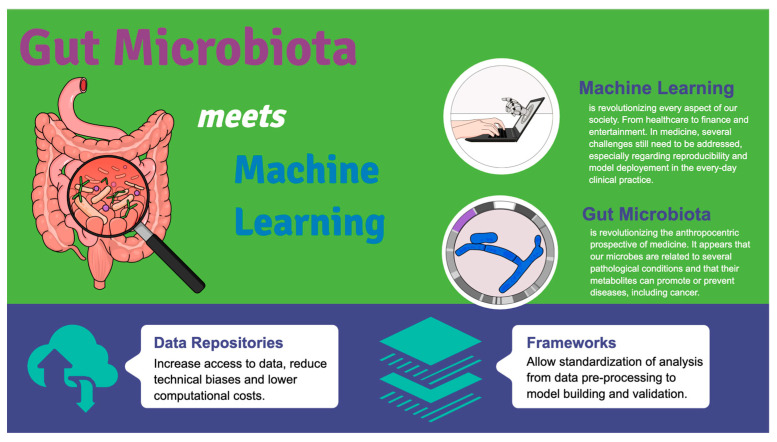
Gut Microbiota meets machine learning. The increasing data availability due to omics analysis has not been followed by the creation of data repositories, guidelines, and analytical frameworks in the past, which resulted in unsatisfactory reproducibility and reliability. The implementation of such tools has facilitated a shift in the field from observational association studies to experimental causal inference and clinical intervention.

From our perspective, computational techniques, particularly machine learning, have played a crucial role in analyzing the large volume of data produced by multi-omics studies of the human gut microbiota, which has led to the discovery of new associations between microbes and disease [10,11,40] as summarized in Figure 1.

In our opinion, the use of machine learning-based analytical processes relies heavily on data availability and requires expertise in implementation to ensure reproducibility and reliability. Fortunately, recent developments in data repositories [29,30,31,32,33], reporting guidelines [34], and frameworks [35,36] have improved the accessibility and transparency of the data analysis process, making it more efficient and reliable. These advancements have facilitated a shift in the field from observational association studies to experimental causal inference and clinical intervention [41]. We believe that this is an exciting development that holds great promise for the future of microbiome research. We anticipate that computational methods will continue to be essential for the analysis of future experimental data [42,43,44] and will drive the development of microbe-based or microbe-directed clinical interventions [24], primarily when used in conjunction with emerging technologies such as cultivation-free genome sequencing [45] and the manipulation of gut microbial genes [46].

In conclusion, we believe that the continued use of computational methods, particularly machine learning, will be critical in advancing our understanding of the complex relationships between the human gut microbiome and disease. We look forward to further developments in this field and anticipate that these advancements will lead to improved clinical interventions and better health outcomes for patients.

## Data Availability

Not applicable.
